# Equid population dynamics in Mexico over five decades: Divergence between national census records and FAOSTAT estimates

**DOI:** 10.1371/journal.pone.0348247

**Published:** 2026-11-05

**Authors:** Elena García-Seco, Miguel Alonso-Díaz, Tamara Tadich, Ramiro E. Toribio, Francisco Galindo, Mariano Hernández-Gil

**Affiliations:** 1 Departamento de Medicina, Cirugía y Zootecnia para Équidos, Facultad de Medicina Veterinaria y Zootecnia, Universidad Nacional Autónoma de México, México City, México; 2 Centro de Enseñanza, Investigación y Extensión en Ganadería Tropical (CEIEGT). Facultad de Medicina Veterinaria y Zootecnia, Universidad Nacional Autónoma de México, Tlapacoyan, Veracruz, México; 3 Programa de Équidos, Instituto de Ciencia Animal, Universidad Austral de Chile, Valdivia, Chile; 4 College of Veterinary Medicine, The Ohio State University, Columbus, Ohio, United States of America; 5 Departamento de Etología, Fauna Silvestre y Animales de Laboratorio, Facultad de Medicina Veterinaria y Zootecnia, Universidad Nacional Autónoma de México, México City, México; Institute of Public Health from Guanajuato State, MEXICO

## Abstract

Equid populations are widely used to inform animal health, welfare, and rural development strategies, yet their interpretation depends on how animals are counted, classified, and reported. Using a repeated cross-sectional analysis of national census records, this study examined long-term changes in Mexico’s equid population and evaluated the consistency between national Agricultural Census records and FAOSTAT livestock statistics. Data for horses, donkeys, and mules were obtained from the Mexican Agricultural Censuses of 1970, 1991, 2007, and 2022, accounting for changes in how equids and their working functions were recorded over time. The total equid population recorded within Mexican Agricultural Production Units — which does not represent Mexico’s overall equid population — declined from 6.82 million animals in 1970 to 1.60 million in 2022, a reduction of 76.5%. Donkeys showed the greatest decline (90.1%), followed by mules (84.1%) and horses (62.0%). In contrast, the census-recorded working-equid population increased by 37.1% between 2007 and 2022, driven predominantly by a 73.0% increase in working horses. Comparison with FAOSTAT revealed a progressively widening discrepancy: the FAOSTAT-to-census ratio increased from 1.82 in 1970 to 8.11 in 2022, with FAOSTAT indicating a 4.5% increase over the same period and observations shifting from official (1970) to estimated (1991, 2007) and imputed (2022). These findings demonstrate a major long-term transformation of Mexico’s equid population while also showing that equids remain functionally important within contemporary agricultural systems, particularly through the increasing contribution of working horses. The marked divergence between national census records and FAOSTAT further highlights the importance of considering census definitions, functional classifications, statistical status, and data provenance when equid population estimates are used for research, veterinary planning, animal-welfare interventions, or public policy.

## Introduction

Equids have played a central role in agricultural production, transport, and rural livelihoods for centuries. Despite widespread mechanization, horses, donkeys, and mules continue to provide transport, packing, riding, traction, and livestock-management functions in many rural production systems [1,2]. Reliable information on their population size, geographical distribution, and functional use is therefore important not only for understanding their changing role in rural systems but also for planning appropriate veterinary services, animal-welfare interventions, and rural-development strategies [3].

The role of equids in agriculture has nevertheless changed substantially with technological and socioeconomic development. Mechanization and motorized transport can replace functions traditionally performed by draft and pack animals, but this transition is not necessarily uniform across production systems. In Mexico, working equids have remained particularly relevant where farmers cannot afford machinery or where topography, farm size, road access, or other physical characteristics limit its practical use [4–6]. Consequently, changes in national equid populations may conceal substantial differences among species, geographical regions, production systems, and types of use.

Understanding these changes requires consideration of how agricultural populations are actually measured. Mexico has a long history of direct agricultural censuses. The National Institute of Statistics and Geography (Instituto Nacional de Estadística y Geografía; INEGI) was created in 1983 and incorporated the functions of the former General Directorate of Statistics (Dirección General de Estadística; DGE), which had been responsible for national census activities since the nineteenth century [7–10]. Unlike livestock population estimates derived primarily from statistical modelling, Mexican Agricultural Censuses are based on direct field enumeration within production units (unidades de producción). This feature allows equid populations to be interpreted in relation to production-unit characteristics and, depending on the census year, to their agricultural or working context.

However, historical population comparisons face a structural challenge: the information collected about equids depended on the structure and objectives of each census questionnaire. Over time, national census instruments altered how equids were classified, moving from explicit working-animal categories to broader species inventories. Consequently, the relationship between an animal recorded by the census and its productive or working function must be interpreted according to the variables available in each census year rather than retrospectively imposing a single contemporary definition of a “working equid.”

This structural limitation is particularly relevant for donkeys and mules. While these species have been historically associated with packing and traction, contemporary populations may perform broader functions, including breeding, companionship, livestock protection, or conservation [11]. Furthermore, recent international concern regarding donkey populations has focused on the global demand for donkey skins used in *ejiao* production [12]. Determining whether recent population changes represent a new phenomenon driven by international trade or form part of a broader, longer-term historical contraction requires population records extending substantially before the expansion of the donkey-skin market and comparison with other equid groups sharing similar agricultural functions.

The interpretation of national equid populations is further complicated by differences among statistical sources. More than two decades ago, de Aluja [5] reported discrepancies among population estimates attributed to Mexican agricultural authorities, INEGI, and the Food and Agriculture Organization of the United Nations (FAO). While the Mexican Agricultural Census obtains information through direct national field enumeration, FAOSTAT compiles standardized country-level time series through international reporting processes, relying on official, estimated, or imputed observations [13]. It is important to note that FAOSTAT data for Mexico rely on official figures reported by INEGI, which are derived from agricultural production units rather than a comprehensive individual-level national census. INEGI censuses record equids only within these production units, so animals kept in equestrian clubs, breeding centers, or non-agricultural households are not captured. Consequently, working equids or animals managed outside formally registered production units may not be fully captured in these time series. Because these sources differ in provenance and methodology, they should not automatically be regarded as interchangeable measures.

The present study had two complementary objectives. First, we examined long-term changes in Mexico’s equid population using Agricultural Census records from 1970, 1991, 2007, and 2022, evaluating changes across species, census classifications, and working functions. Second, we compared total equid populations recorded by the Mexican Agricultural Census with corresponding FAOSTAT records for the same census years and examined the statistical status associated with FAOSTAT observations to quantify discrepancies and evaluate the importance of data provenance in livestock statistics.

## Materials and methods

### Study design and data sources

A repeated cross-sectional study was conducted to characterize changes in Mexico’s equid population and to evaluate the consistency between national agricultural census records and international livestock statistics. The study comprised two complementary objectives. The first examined long-term changes in the national equid population and in the population of working equids within production units using data from the Mexican Agricultural Censuses conducted by the National Institute of Statistics and Geography (Instituto Nacional de Estadística y Geografía; INEGI). The second compared total equid population records from INEGI with corresponding observations reported in the Food and Agriculture Organization Corporate Statistical Database (FAOSTAT), including the statistical status associated with FAOSTAT observations.

### INEGI population data and working-equid classification

For the first objective, data were obtained from the Mexican Agricultural Censuses of 1970, 1991, 2007, and 2022, spanning a 52-year period. The 1970 census was conducted by the former General Directorate of Statistics (*Dirección General de Estadística*; DGE), whereas the 1991, 2007, and 2022 censuses were conducted by the National Institute of Statistics and Geography (*Instituto Nacional de Estadística y Geografía*; INEGI). Data were aggregated at the level of subnational administrative divisions. In this study, the term ‘federal entities’ (*entidades federativas*) is used instead of ‘states’ to formally include both the 31 states and Mexico City, following the official geopolitical classification of the National Institute of Statistics and Geography (INEGI). Historical census records for 1970 were published by the DGE as a series of federal entity volumes [7]; each of the 31 federal entity and Mexico City volumes were consulted individually, and national totals were obtained by manually aggregating the federal entity figures. Records for 1970 and 1991 were consulted from original printed census publications held at the Public Library of the Institute of Geography, National Autonomous University of Mexico (*Universidad Nacional Autónoma de México*; UNAM). Census publications, questionnaires, methodological documents, and electronic tabulations available for the more recent censuses were retrieved from the official INEGI website (https://www.inegi.org.mx). Population data were manually extracted from the corresponding census tables and compiled by species and census year for analysis.

Two population measures were considered: total equid population and working-equid population within production units. Total equid population was calculated as the sum of horses, donkeys, and mules recorded in each census. Data reported under ‘mules’ includes both mules and hinnies, as official INEGI census figures do not separate these hybrids. Because the criteria used to classify equids differed among Agricultural Censuses, the working-equid population was identified according to the categories provided in each census rather than by applying a single classification retrospectively across all four census years.

In the 1970 Agricultural-Livestock Census, livestock inventories included an explicit category of “working animals”, defined by the census as large livestock used in agricultural activities. Working-equid counts were therefore obtained from the corresponding census categories for animals used for agricultural work.

In the 1991 Agricultural Census, equid inventories were classified by type of production unit, including urban and rural production units, as well as by land tenure and production-unit size. For the purposes of this study, equids recorded within rural production units were used to characterize the working-equid population. This represents an operational classification based on the rural agricultural context in which the animals were recorded rather than an individual assessment of the specific task performed by each animal.

For the 2007 and 2022 censuses, working-equid populations were identified using the categories available in the corresponding census records. In 2022, horses were explicitly separated into animals kept for recreational purposes and those classified as “for riding or work”; only the latter were included as working horses. Donkeys and mules were recorded as single species categories without an equivalent functional subdivision. In 2007, horses, donkeys, and mules were recorded within the rural production-unit framework, but horses were not subdivided into recreational and riding-or-work categories as in 2022. Therefore, the operational definition of working equids followed the classification available in each census year.

Because functional classification and the structure of the census records differed among years, long-term working-equid estimates were interpreted within the context of the classification available in each census. **Federal-entity-level** population counts from the 2022 Agricultural Census were compiled for each of Mexico’s 32 federal entities (31 states and Mexico City). Choropleth maps were generated using vector boundaries from the INEGI *Marco Geoestadístico* (https://www.inegi.org.mx/programas/mg/), which were adapted by the authors and linked to the corresponding census counts by federal entity..

### Comparison with FAOSTAT

For the second objective, FAOSTAT livestock population records for Mexico were retrieved for the same four census years (1970, 1991, 2007, and 2022), thereby allowing direct temporal comparison with the INEGI census series. FAOSTAT records for horses, asses, and hybrids (mules and hinnies) were extracted, and the three categories were summed to obtain the total equid population for each year. For readability and consistency with INEGI terminology, we use the common terms ‘donkeys’ and ‘mules’ throughout to refer to FAOSTAT’s ‘Asses’ and ‘Mules and hinnies’ categories, respectively.The FAOSTAT dataset used for the final analysis was downloaded on 8 August 2026.

Comparisons between FAOSTAT and INEGI were performed using total equid inventories rather than the INEGI working-equid subset, because the FAOSTAT livestock population series does not provide an equivalent functional classification separating working from non-working equids.

The metadata flag associated with each FAOSTAT observation was retained as part of the dataset. For the census years examined, FAOSTAT observations were identified as official (A), estimated (E), or imputed (I), according to the status assigned in the downloaded database. These classifications were analyzed descriptively together with the magnitude of the discrepancy between FAOSTAT and INEGI.

### Statistical analysis

Population changes were evaluated using descriptive and comparative analyses. For each equid category, absolute and percentage changes between census years were calculated. Because the intervals between Agricultural Censuses were unequal, the average annual variation rate (AAVR) was calculated using a geometric rate of change:


$$\begin{array}{c} AAVR=\left[{\left(\frac{Vfinal}{Vinitial\;}\right)}^{\frac{\text{1}}{t}}-\text{1}\right]X\;\text{1}\text{0}\text{0} \end{array}$$

where *V final =* population of the most recent census, *V initial* = population of the previous census, and *t* = number of years between censuses [14]. This measure was used to compare the direction and magnitude of population change across census intervals of different lengths.

A sensitivity analysis was conducted to assess the robustness of population-change estimates to potential uncertainty in historical census counts. Census values were varied independently by ±5% and ±10% at the beginning and end of each comparison interval, and the resulting range of possible percentage changes was calculated. A population trend was considered directionally robust when the sign of the estimated change remained unchanged across the corresponding perturbation range. This analysis was intended to distinguish large, robust long-term population changes from small changes that could be sensitive to modest variation in the underlying census counts.

Changes in species composition were evaluated using chi-square tests of independence based on census counts of horses, donkeys, and mules. Because the very large census populations could produce statistically significant results even for relatively small proportional differences, effect size was quantified using Cramér’s V. Comparisons were performed for 1970 versus 2022 to assess long-term compositional change and for 2007 versus 2022 to characterize more recent changes in population structure. Statistical significance was set at P < 0.05. All calculations, including the AAVR, sensitivity analysis, chi-square tests, and Cramér’s V, were performed in Microsoft Excel for Mac, version 16.112.2 (Microsoft 365 subscription; Microsoft Corporation, Redmond, WA, USA).

For the geographical analysis of working equids, absolute and percentage changes between 2007 and 2022 were calculated for each of Mexico’s federal entities. Changes were evaluated separately for horses, donkeys, and mules, as well as for the combined working-equid population. Federal entities count analyses were descriptive and were used to characterize the magnitude and geographical distribution of population change.

For the comparison between INEGI and FAOSTAT, total equid populations were calculated for each corresponding census year (1970, 1991, 2007, and 2022).

The absolute difference between sources was calculated as FAOSTAT − INEGI; the relative difference was calculated as [(FAOSTAT − INEGI)/INEGI] × 100; and the FAOSTAT-to-INEGI ratio was calculated as FAOSTAT/INEGI.

Long-term percentage change from 1970 to 2022 was calculated independently for each data source to compare the population trajectories described by the two statistical series. FAOSTAT data-status flags (official, estimated, or imputed) were examined descriptively in relation to the magnitude of the observed discrepancies; no inferential test or causal relationship between data status and source divergence was assumed.

## Results

### Objective 1. Long-term population dynamics and geographical distribution of equids in Mexico

The structural characteristics and recording criteria for equids varied across the four national censuses evaluated. While the 1970 census explicitly recorded working animals, subsequent censuses transitioned to production-unit species inventories (1991 and 2007) and, most recently, introduced functional subdivisions exclusively for horses (2022). Based on these census-specific classifications, long-term total population dynamics and working-equid trends were evaluated (Table 1).

**Table 1:** Evolution of census architecture, equid classification criteria, and operational definitions of working equids in Mexican Agricultural Censuses (1970–2022).

Census	Responsible Institution	Primary survey unit	Equid classification and recording	Operational Definition of Working Equids in this study
**1970**	General Directorate of Statistics (DGE)	Agricultural-livestock holdings (*predios*)	Explicit species inventory separating horses, donkeys, and mules. Included an explicit, dedicated category for “working animals” used in agricultural activities.	Equids explicitly recorded under the “working animals” category in agricultural holdings.
**1991**	National Institute of Statistics and Geography (INEGI)	Rural Production Units	Reported as horses, donkeys, and mules within production units. Disaggregated by rural/urban location, land tenure, and farm size (≤5 ha and >5 ha), but without explicit species-specific work categories.	Equids recorded within rural production units (contextual operational definition).
**2007**	National Institute of Statistics and Geography (INEGI)	Rural Production Units	Equids recorded under “Other animal species” as overall species totals without species-specific functional or zootechnical subdivisions.	All equids recorded within rural production units (operational definition based on agricultural context).
**2022**	National Institute of Statistics and Geography (INEGI)	Agricultural Production Units	Equids recorded under “Other animal species”. Introduced an explicit functional subdivision for horses: “for recreational purposes” vs. “for riding or work”. Donkeys and mules remained as single species categories.	Horses explicitly classified as “for riding or work”, plus all donkeys and mules within agricultural production units.

**Note**: DGE = Dirección General de Estadística; INEGI = Instituto Nacional de Estadística y Geografía. Data for 1970 were retrieved from printed federal entity volumes held at the UNAM Institute of Geography Library [7]. Data for 1991, 2007, and 2022 were retrieved from official INEGI census publications and electronic databases [8–10].

### Long-term changes in Mexico’s equid population, 1970–2022

The equid population recorded within Mexican Agricultural Production Units declined markedly over the 52-year study period. The total population recorded by INEGI within these production units decreased from 6,820,159 equids in 1970–1,603,119 in 2022, representing an overall reduction of 76.5%. The decline was progressive across successive Agricultural Censuses, with 5,180,707 equids recorded in 1991 and 2,143,934 in 2007 (Fig 1).

**Fig 1:**
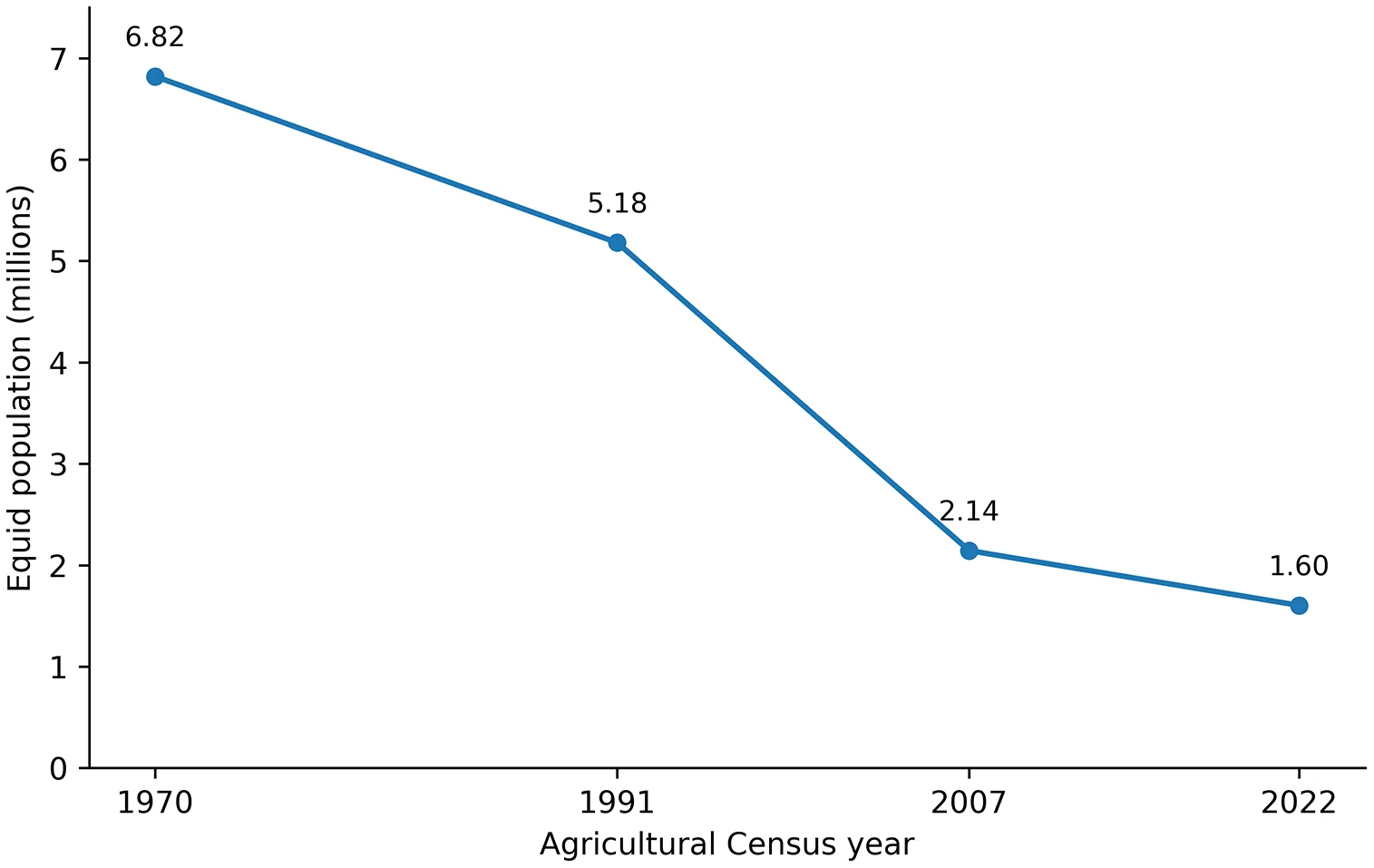
Long-term change in the equid population recorded by the Mexican Agricultural Censuses, 1970–2022. Total equid population recorded by the National Institute of Statistics and Geography (INEGI) in the Agricultural Censuses of 1970, 1991, 2007, and 2022. The census-recorded population decreased from 6.82 million equids in 1970 to 1.60 million in 2022, representing an overall reduction of 76.5% during the 52-year study period.

The magnitude of long-term change differed among equid groups. Donkeys showed the greatest reduction between 1970 and 2022, declining from 2,893,525–287,039 (−90.1%), followed by mules, which declined from 792,259–125,854 (−84.1%). Horses showed a less pronounced decline, from 3,134,375–1,190,226 (−62.0%), resulting in a substantial change in the species composition of the national equid population over the study period (Fig 2).

**Fig 2:**
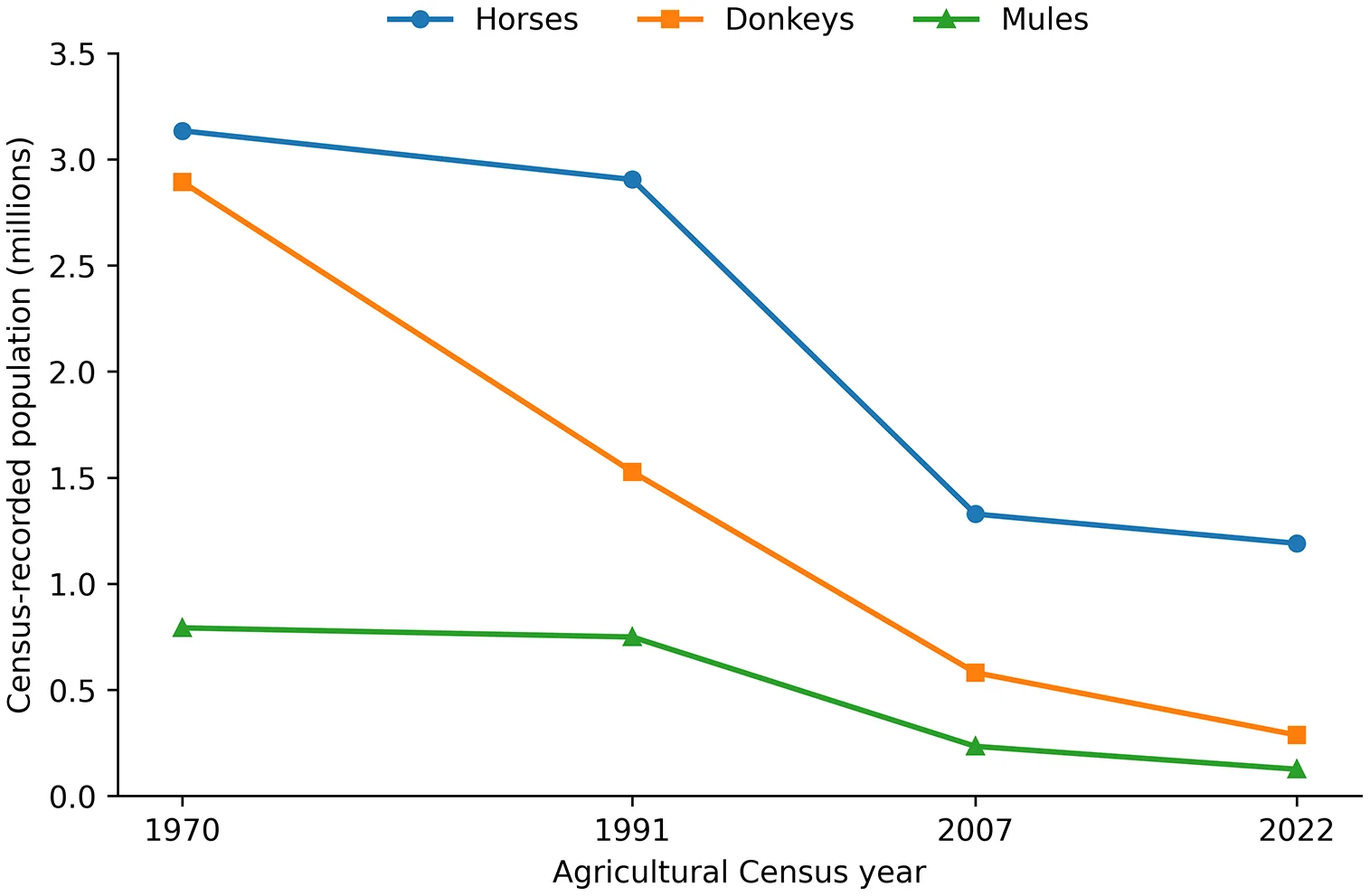
Long-term changes in census-recorded horse, donkey, and mule populations in Mexico, 1970–2022. Population counts recorded in the Mexican Agricultural Censuses of 1970, 1991, 2007, and 2022 are shown separately for horses, donkeys, and mules. Between 1970 and 2022, horses declined from 3.13 to 1.19 million (−62.0%), donkeys from 2.89 million to 0.29 million (−90.1%), and mules from 0.79 million to 0.13 million (−84.1%).

### Changes in species composition

Changes in population size were accompanied by a marked shift in species composition. The distribution of horses, donkeys, and mules differed significantly between 1970 and 2022 (χ^2^ = 666,425, df = 2, P < 0.001), with a Cramér’s V of 0.331, indicating a substantial long-term change in population structure. A significant but smaller compositional change was also detected between 2007 and 2022 (χ^2^ = 46,041, df = 2, P < 0.001; Cramér’s V = 0.144).

### Sensitivity analysis

The sensitivity analysis indicated that the major long-term population declines were robust to perturbation of the census counts. The direction of the 1970–2022 declines remained unchanged under both the ± 5% and ±10% scenarios. In contrast, the relatively small changes observed for donkeys and mules between 2007 and 2022 were sensitive to perturbation. Under the ± 10% scenario, the estimated change ranged from −22.4% to +15.9% for donkeys and from −21.5% to +17.3% for mules. Thus, although the observed census counts indicated small declines in both groups during the most recent period, the direction of these changes was not robust to modest variation in the underlying counts (Table 2).

**Table 2:** Sensitivity of equid population-change estimates to perturbation of census counts, 1970–2022 and 2007–2022.

Period	Population	Observed change (%)	Change under ±5% perturbation (%)	Change under ±10% perturbation (%)	Direction robustness
**1970–2022**	Total equids	−76.5	−78.7 to −74.0	−80.8 to −71.3	Yes
	Horses	−62.0	−65.6 to −58.0	−68.9 to −53.6	Yes
	Donkeys	−90.1	−91.0 to −89.0	−91.9 to −87.9	Yes
	Mules	−84.1	−85.6 to −82.4	−87.0 to −80.6	Yes
**2007–2022**	Donkeys	−5.2	**−14.2 to +4.8**	**−22.4 to +15.9**	**No**
	Mules	−4.1	**−13.2 to +6.0**	**−21.5 to +17.3**	**No**

**Note:** Sensitivity analysis was performed by independently perturbing the population counts at the beginning and end of each comparison period by ±5% and ±10%. Reported ranges represent the minimum and maximum percentage changes obtained under the corresponding perturbation scenarios. The direction of change was considered robust when all scenarios retained the same direction as the observed change. Because donkeys and mules showed comparatively small changes between 2007 and 2022, their perturbation ranges crossed zero, indicating that these directional changes are more sensitive to measurement uncertainty than the larger, long-term declines observed between 1970 and 2022.

### Changes in the census-recorded working-equid population, 2007–2022

Between 2007 and 2022, the census-recorded population classified in this study as working equids increased from 941,643–1,291,147 animals, corresponding to an increase of 37.1%. This change was driven predominantly by horses, whose population within the corresponding working-equid categories increased from 507,670–878,254 (+73.0%). In contrast, donkeys decreased from 302,779–287,039 (−5.2%), while mules decreased from 131,194–125,854 (−4.1%). Consequently, horses accounted for an increasing proportion of the census-recorded working-equid population during this period (Table 3).

**Table 3:** Changes in census-recorded working-equid populations in Mexico between 2007 and 2022.

Working-equid category	2007	2022	Absolute change	Change (%)
Horses for riding or work	507,670	878,254	+370,584	**+73.0%**
Donkeys	302,779	287,039	−15,740	**−5.2%**
Mules	131,194	125,854	−5,340	**−4.1%**
**Total working equids**	**941,643**	**1,291,147**	**+349,504**	**+37.1%**

*Note:* Working equids were operationally defined using the census categories available in each census year, as described in Materials and Methods. For the purposes of the 2007–2022 comparison, the population comprised horses included within the corresponding working-equid categories, together with donkeys and mules. Because census classification differed between years, comparisons should be interpreted as changes in census-recorded working-equid populations rather than as changes based on an identical individual-level functional classification. Absolute change was calculated as the 2022 population minus the 2007 population, and percentage change relative to the 2007 population.

### Geographical patterns of working-equid change

The increase in census-recorded working equids between 2007 and 2022 was geographically widespread (Table 4). Total working-equid populations increased in 29 of Mexico’s 32 federal entities, with decreases observed only in Guanajuato, Quintana Roo, and Colima. The horse component increased in 31 of 32 federal entities, whereas donkey and mule populations showed more heterogeneous geographical patterns, increasing in 12 and 9 federal entities, respectively, and declining in the remaining federal entities.

**Table 4:** Federal entity-level distribution and changes in census-recorded working-equid populations in Mexico, 2007–2022.

A. Federal entities with the largest working-equid populations in 2022				
**Rank**	**Federal entity**		**Working equids (2022)**	
1	Guerrero		164,125	
2	Veracruz		111,577	
3	Puebla		106,484	
4	Oaxaca		87,369	
5	Estado de Mexico		76,349	
6	San Luis Potosi		73,336	
7	Chiapas		56,749	
8	Durango		55,250	
9	Michoacan		55,036	
10	Chihuahua		54,440	
11	Zacatecas		53,423	
12	Guanajuato		49,630	
**B. Federal entities with decreases in total working-equid populations between 2007 and 2022**				
**Federal entity**	**2007**	**2022**	**Change**	
Quintana Roo	981	927	−5.5%	
Guanajuato	51,604	49,630	−3.8%	
Colima	3,294	3,247	−1.4%	
**C. Largest absolute increases in the horse component of the working-equid population between 2007 and 2022**				
**Federal entity**	**2007**	**2022**	**Absolute increase**	**Percentage change**
Guerrero	40,114	81,271	+41,157	+102.6%
Puebla	34,455	63,018	+28,563	+82.9%
San Luis Potosí	24,049	52,311	+28,262	+117.5%
Veracruz	52,622	80,642	+28,020	+53.2%
Estado de Mexico	34,666	56,125	+21,459	+61.9%
Zacatecas	20,712	41,165	+20,453	+98.7%
Durango	20,842	40,978	+20,136	+96.6%

*Note:* Working equids were operationally defined using the corresponding census categories described in Materials and Methods. Panel A shows the federal entities with the largest total working-equid populations in 2022; Panel B shows the federal entities in which the combined working-equid population decreased between 2007 and 2022; Panel C shows the largest absolute increases in the horse component of the working-equid population during the same period.

Guerrero had the largest census-recorded working-equid population in 2022 (164,125), followed by Veracruz (111,577), Puebla (106,484), Oaxaca (87,369), Estado de Mexico (76,349), and San Luis Potosí (73,336). Guerrero was also notable for increases in all three groups: horses increased from 40,114 in 2007–81,271 in 2022, donkeys from 50,741–61,254, and mules from 17,433–21,600.

### Regional distribution of working equids

Regional aggregation revealed marked geographical differences in the distribution and temporal change of working equids between 2007 and 2022. The regional pattern was driven primarily by changes in the horse component of the working-equid population, whereas donkey and mule populations showed smaller and more heterogeneous changes (Fig 3).

**Fig 3:**
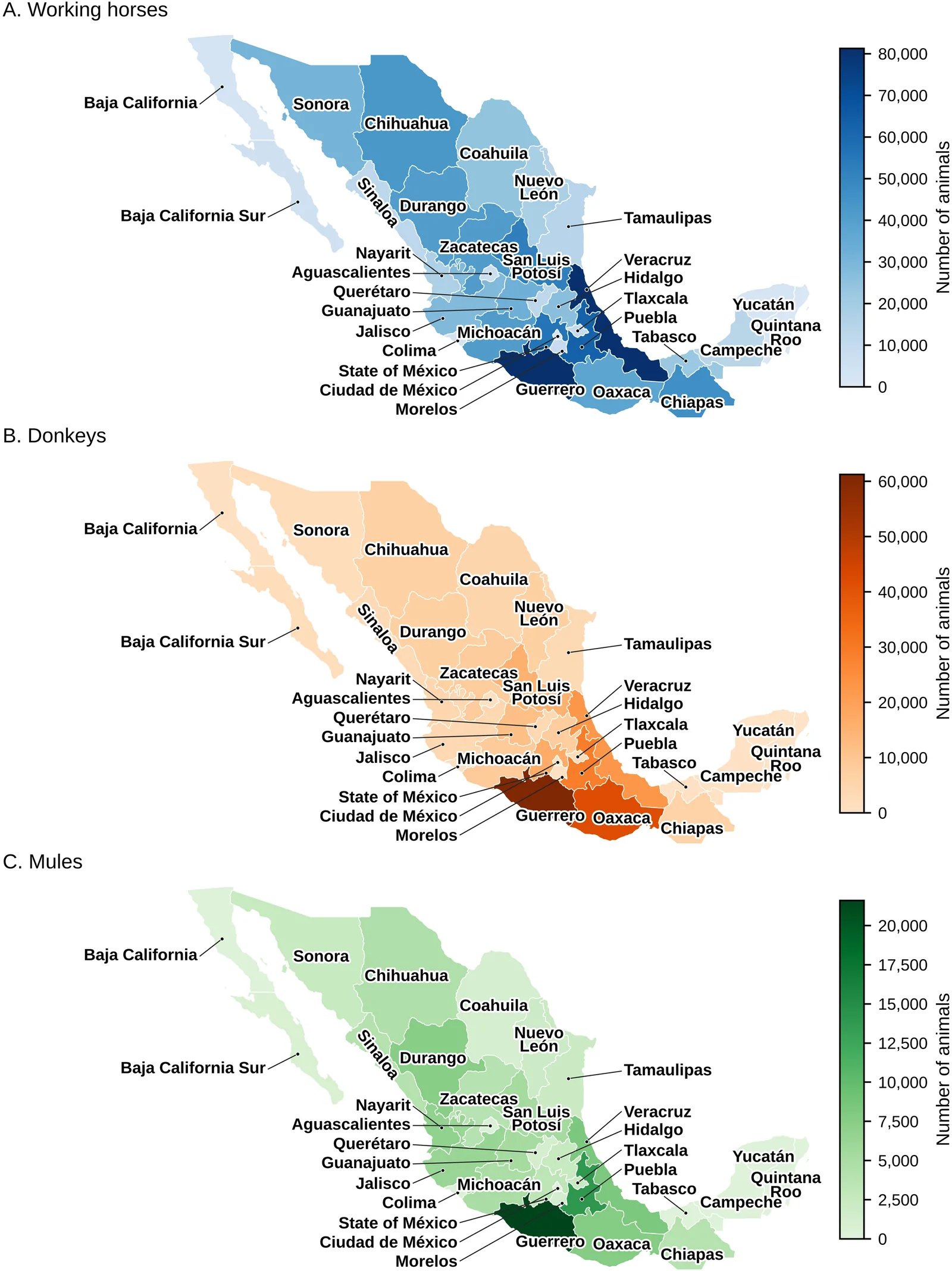
Geographical distribution of working equids in Mexico in 2022. Federal entities distribution of (A) working horses, (B) donkeys, and (C) mules recorded in the 2022 Agricultural Census. Color intensity represents the number of animals recorded in each federal entity. Geographic boundaries were obtained from the INEGI *Marco Geoestadístico, Censo Agropecuario 2022* and adapted by the authors for visualization. Labels are shown for the 12 federal entities with the largest total working-equid populations in 2022 (Table 4, Panel A); the remaining federal entities are represented by color only.

### Objective 2. Divergence between INEGI and FAOSTAT population records

Marked and progressively increasing differences were observed between total equid populations recorded by the INEGI Agricultural Censuses and those reported by FAOSTAT for the corresponding census years (Table 5). In 1970, FAOSTAT reported 12,434,559 equids compared with 6,820,159 recorded by INEGI, corresponding to a FAOSTAT-to-INEGI ratio of 1.82 and a relative difference of +82.3%. The discrepancy increased in subsequent census years: in 1991, FAOSTAT reported 12,553,000 equids compared with 5,180,707 recorded by INEGI (2.42-fold; + 142.3%), and in 2007, 12,890,000 compared with 2,143,934 (6.01-fold; + 501.2%). By 2022, FAOSTAT reported 12,995,724 equids compared with 1,603,119 recorded by INEGI, corresponding to an 8.11-fold difference (+710.7%).

**Table 5:** Comparison of total equid populations recorded by INEGI and reported by FAOSTAT for corresponding Mexican Agricultural Census years, 1970–2022.

Census year	INEGI total equids	FAOSTAT total equids	Absolute difference	FAOSTAT/INEGI ratio	Difference relative to INEGI	FAOSTAT data status
1970	6,820,159	12,434,559	5,614,400	1.82	+82.3%	Official (A)
1991	5,180,707	12,553,000	7,372,293	2.42	+142.3%	Estimated (E)
2007	2,143,934	12,890,000	10,746,066	6.01	+501.2%	Estimated (E)
2022	1,603,119	12,995,724	11,392,605	8.11	+710.7%	Imputed (I)

Note*:* Absolute difference = FAOSTAT − INEGI. Relative difference = [(FAOSTAT − INEGI)/INEGI] × 100. FAOSTAT data status corresponds to the metadata accompanying each observation: A = official; E = estimated; I = imputed. FAOSTAT data were downloaded on 8 August 2026.

### Contrasting long-term trajectories

In addition to the increasing difference in population magnitude, the two data sources described contrasting long-term trajectories. Between 1970 and 2022, the total equid population recorded by INEGI decreased by 76.5%, from 6.82 million to 1.60 million animals. In contrast, the corresponding FAOSTAT population increased by 4.5%, from 12.43 million to 13.00 million. Consequently, the divergence between the two series widened progressively over the study period, from an absolute difference of 5.61 million equids in 1970 to 11.39 million in 2022.

### FAOSTAT data status

The statistical status associated with the FAOSTAT observations also differed across census years. The 1970 observations were classified as official (A), those corresponding to 1991 and 2007 as estimated (E), and those for 2022 as imputed (I). The largest difference between the two data sources occurred in 2022, when the FAOSTAT observations for horses, donkeys, and mules were all classified as imputed. The association between increasing source divergence and changes in FAOSTAT data status was examined descriptively; no causal relationship was inferred (Fig 4).

**Fig 4:**
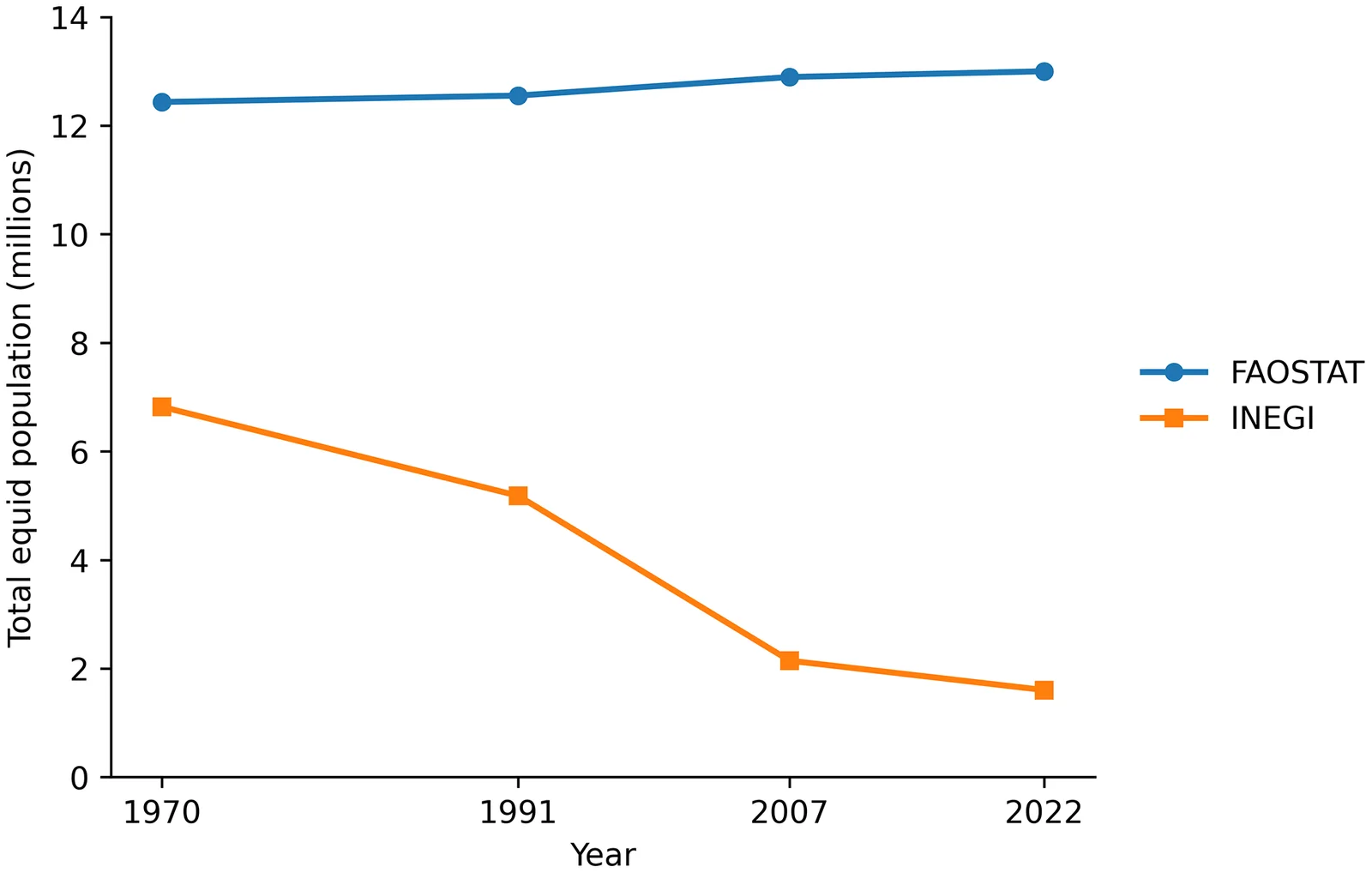
Contrasting long-term trajectories of equid populations reported by INEGI and FAOSTAT for Mexico, 1970–2022. Total equid populations were calculated as the sum of horses, donkeys, and mules reported by each data source for the corresponding census years. INEGI census records show a marked decline over the study period, whereas FAOSTAT values remain comparatively stable.

## Discussion

### Principal findings and historical context

This study provides a 52-year assessment of equid population dynamics in Mexico and reveals two major structural findings. First, national census data document a profound long-term contraction of Mexico’s total equid population (76.5% decline between 1970 and 2022), with donkeys (−90.1%) and mules (−84.1%) experiencing the most severe reductions. However, the most recent intercensal period (2007–2022) revealed a striking functional shift: the census-recorded working-equid population increased by 37.1%, driven predominantly by a 73.0% increase in horses classified “for riding or work” across 31 of 32 federal entities.

Second, comparison with international statistics revealed a progressively widening divergence between national census counts and FAOSTAT figures, increasing from a 1.82-fold discrepancy in 1970 to an 8.11-fold difference by 2022. Crucially, the two sources describe opposing demographic trajectories: while INEGI documents a 76.5% decline over 52 years, FAOSTAT indicates a slight 4.5% increase. This divergence coincided with shifts in FAOSTAT statistical status flags from official (1970) to estimated (1991, 2007) and imputed (2022) observations, underscoring the critical impact of data provenance on livestock accounting [15,16].

### Long-term demographic decline vs. recent international pressures

The 52-year timeframe demonstrates that the contraction of Mexico’s equid population was established long before the emergence of the contemporary global donkey-skin trade for *ejiao* production [12,17,18]. Total equid numbers declined continuously across the 1970, 1991, and 2007 censuses. Because the most drastic reductions in donkey populations occurred prior to the expansion of the international skin trade in the 2010s [12,17,18], this trade cannot be considered the primary driver of the historical decline.

This conclusion is reinforced by the parallel long-term trajectory observed in mules (−84.1% between 1970 and 2022). Mules and donkeys historically fulfilled similar agricultural roles in packing, transport, and traction [2], yet mules are not targeted by the *ejiao* industry [12]. The shared decline of both species points to broader, structural transformations in rural Mexico, most notably agricultural mechanization and the expansion of motorized transport infrastructure [6,19]. As mechanical power replaced animal traction, functional demand for both species decreased. Furthermore, because mule breeding depends directly on donkey availability [20], the sustained contraction of breeding donkeys likely reinforced the decline in mule production over time.

### The 2007–2022 shift and persistent relevance of working horses

The increase in working equids between 2007 and 2022 (+37.1%) does not indicate a reversal of the historical overall decline, but rather a compositional shift within Agricultural Production Units. This increase was driven almost entirely by horses classified “for riding or work” (+73.0%), whereas recent changes in donkeys (−5.2%) and mules (−4.1%) were small and, as shown by the sensitivity analysis, directionally sensitive to minor count perturbations.

The widespread increase in working horses—occurring across 31 federal entities—challenges linear models assuming that agricultural modernization inevitably leads to the total disappearance of draft animals [6]. While tractors displace animal traction in tillage, horses maintain functional utility in cattle management, herd monitoring, personal mobility, and transport in rugged terrains where motor vehicles remain impractical [21,22]. This pattern reflects functional specialization rather than technological obsolescence. High concentrations of working equids in federal entities such as Guerrero, Veracruz, Puebla, Oaxaca, Estado de Mexico, and San Luis Potosi highlight their enduring importance in smallholder livelihoods and complex topographies [1,5,23,24].

### Divergence between national censuses and FAOSTAT: Implications for policy and welfare

The expanding discrepancy between INEGI census counts (1.60 million equids in 2022) and FAOSTAT figures (12.99 million in 2022) has profound implications for applied decision-making. A gap exceeding 11 million animals alters the baseline denominator used to evaluate animal health coverage, disease risk, welfare intervention scale, and veterinary resource allocation [25,26].

While imputation procedures in global databases like FAOSTAT are necessary to maintain continuous country time series when primary reporting is absent [16], the uncritical use of imputed figures risks compounding error. When secondary users treat imputed figures as direct observational counts without reporting status flags, inaccurate population baselines become embedded in public policy and academic literature [27].

For working equids, accurate population planning requires more than updating national totals. It requires distinguishing species, functional roles, and subnational distributions [1,26,28]. A single aggregated species total cannot capture whether animals are used for transport, traction, or recreation, nor can it identify regional welfare priorities. Improving population estimates therefore depends on maintaining transparency regarding data provenance, incorporating metadata flags, and relying on primary national census frameworks that capture the agricultural context of working animals.

The magnitude of the divergence documented here — an 8.11-fold difference by 2022 — is specific to Mexico’s census infrastructure and cannot be directly extrapolated to other countries or livestock categories, since the size of any INEGI-FAOSTAT-type gap depends on country-specific census frequency, methodology, and reporting practices. However, the underlying structural pattern is plausibly more general: FAOSTAT’s reliance on official national reporting means that, as the interval since the last primary census lengthens, its data-status classification for a given country and species shifts progressively from official to estimated to imputed, potentially amplifying the very divergence it is meant to smooth over. This pattern is not unique to Mexican equids and may affect any country or livestock category where recent primary national census data are unavailable, underscoring the broader relevance of transparent data-status reporting in global livestock statistics beyond the specific case examined here.

### Study limitations

A key limitation of this study concerns the scope of the Mexican Agricultural Census itself. INEGI censuses enumerate equids exclusively within production units, and animals kept outside this agricultural framework—including those in equestrian clubs, breeding centers, urban stables, or non-agricultural households—are not captured. Consequently, the population figures reported here should not be interpreted as the total national equid population, but as the census-recorded equid population within production units, which represents the only population that can be measured systematically and consistently across the 1970–2022 period.

A second limitation concerns the operational definition of “working equids” used in this study. The criteria used to identify working equids differed across census years (Table 1). In 1970, the census included an explicit, dedicated “working animals” category. In 1991 and 2007, no equivalent species-specific functional category existed; instead, working equids were approximated as those recorded within rural production units, based on the assumption that equids counted in this rural framework were predominantly used for work — a contextual, rather than an explicit, operational definition. In 2022, INEGI introduced a different approach, explicitly subdividing horses into those “for riding or work” and those kept “for recreational purposes” within Agricultural Production Units, which, unlike the 1991 and 2007 rural-only frame, were not restricted to rural areas. Because these three classification mechanisms — an explicit category in 1970, a rural/urban proxy in 1991 and 2007, and an explicit functional subdivision within a broader survey frame in 2022 — are not equivalent, the working-equid variable does not represent an identical, individual-level functional classification across census years. Consequently, the apparent increase in the working-equid population between 2007 and 2022 partly reflects this change in census methodology and classification criteria, rather than a strictly comparable measurement of an identical underlying category, and comparisons across census years should be interpreted with this structural change in mind.

## Conclusions

Mexico’s equid population has undergone a profound demographic transformation over the past five decades. Mexican Agricultural Census data indicate a 76.5% reduction in the total equid population between 1970 and 2022, with particularly pronounced long-term declines in donkeys (90.1%) and mules (84.1%). The timing and parallel nature of these declines indicate that recent demand for donkey skins cannot, by itself, explain the historical contraction and instead support consideration of broader transformations in the use and demand for traditional working equids.

At the same time, recent census data demonstrate that working equids continue to play a substantial role within Mexican agricultural systems. Between 2007 and 2022, the census-recorded working-equid population increased by 37.1%, driven predominantly by a 73.0% increase in working horses, classified by INEGI as horses “for riding or work”. This increase was geographically widespread, with working-horse populations increasing in 31 of Mexico’s 32 federal entities. These findings suggest that the role of equids within contemporary agricultural systems is changing rather than simply disappearing and highlight the importance of distinguishing species and functional categories when assessing equid population dynamics.

Finally, the progressive divergence between Mexican Agricultural Census records and FAOSTAT demonstrates the importance of data provenance in population assessments. By 2022, the FAOSTAT total was 8.11 times the corresponding Mexican Agricultural Census count, and the two sources described contrasting long-term demographic trajectories: a 76.5% decline in the census series compared with a 4.5% increase in FAOSTAT. Population estimates should therefore be interpreted together with their definitions, statistical status, and source methodology, particularly when they are used to inform research, veterinary planning, animal-welfare interventions, or public policy.
