# Supplementary material for: Equid population dynamics in Mexico over five decades: Divergence between national census records and FAOSTAT estimates
Source: PLoS One. 2026 Nov 5;21(11):e0348247. doi: 10.1371/journal.pone.0348247 (PMC13739109; doi:10.1371/journal.pone.0348247)
Supplement: Supplementary file 1 [file pone.0348247.s002.docx]

**Response to Reviewers**

*Manuscript: "Equid population dynamics in Mexico over five decades: Divergence between national census records and FAOSTAT estimates"*

We thank the Academic Editor and both reviewers for their careful and constructive evaluation of our manuscript. In response to their comments, the manuscript has been substantially rewritten. Below we summarize the scope of that revision, then respond point by point to every comment raised in the review. A version of the manuscript with tracked changes against the originally submitted version is included as a separate file with this resubmission.

**Summary of the revision**

Because the reviewers' concerns touched on nearly every part of the manuscript — statistical rigor, methodological justification, internal consistency, data availability, and language — we chose to rewrite the manuscript rather than patch it incrementally. The principal changes are:

- The title and framing were changed from an evaluative framing ("Statistical invisibility of working equids in Mexico...") to a neutral, descriptive one ("Equid population dynamics in Mexico over five decades: Divergence between national census records and FAOSTAT estimates").
- The adapted IUCN Red List classification was removed in its entirety and replaced with a formal chi-square test of independence and Cramér's V effect size for compositional change, plus a sensitivity analysis (±5%/±10%) addressing census counting error.
- The FAOSTAT–INEGI comparison was extended from a single year (2022) to all four census years (1970, 1991, 2007, 2022), and FAOSTAT's own data-status flags (official/estimated/imputed) are now reported and discussed.
- All causal and evaluative language flagged by Reviewer #1 ("staggering," "drastic," "resilience of necessity," "paradox of modernization," "bio-cultural erosion," etc.) was removed; undefined conceptual terms were either formally defined or eliminated.
- The horse-meat-trade section was removed, as it was not integrated into the quantitative analysis.
- A formal Data Availability Statement was added to the manuscript body, an OSF repository was prepared with the full analytical dataset (including the complete 32-federal-entity 2022 breakdown), and a STROBE checklist is now submitted as supplementary material (S1).
- All in-text citations were converted to PLOS ONE's numbered format, and the reference list was expanded and re-verified, including the addition of primary citations for the 1991 and 2007 Agricultural Censuses.
- The choropleth map figure was rebuilt from non-copyrighted boundary data obtained directly from the INEGI Marco Geoestadístico, with correct federal-entity labels, replacing the original figures flagged for possible copyright issues.
- Funding information was removed from the Acknowledgments section; the corrected Funding Statement is provided at the end of this letter.

Because of the scope of this rewrite, several of the reviewers' line-level textual corrections (e.g., specific word replacements in since-removed sentences) no longer apply to specific sentences — the underlying language issue they identify has been resolved throughout the manuscript rather than at the original location. We note this explicitly where relevant below.

**Editor / Journal Requirements**

***Reviewer comment:*** *Thank you for stating funding information in the Acknowledgments Section... Please remove any funding-related text from the manuscript and let us know how you would like to update your Funding Statement.*

**Response:** The funding-related text has been removed from the Acknowledgments section, which now lists only the student contributors (Luis Eduardo Molina Corona, Javier Beltrán Romero, Erick Zuñiga Martínez, and María Fátima Rodríguez Vargas). The corrected Funding Statement, to be entered in the online submission system, is: "This work was not supported by a specific research grant. E.G.S. received a PASPA fellowship (Programa de Apoyo a la Superación del Personal Académico, DGAPA-UNAM) during the period of this study, as part of doctoral studies. The funder had no role in study design, data collection and analysis, decision to publish, or preparation of the manuscript."

***Reviewer comment:*** *We note that Figures 1, 7, 8 and 9 in your submission contain map images which may be copyrighted... We require you to either (1) present written permission from the copyright holder... or (2) remove the figures.*

**Response:** The choropleth map figure was rebuilt using state boundaries obtained directly from INEGI's own Marco Geoestadístico (https://www.inegi.org.mx/programas/mg/), the same official geographic boundary dataset cited in the manuscript's Materials and Methods, not from any proprietary or copyrighted map service. The figure now includes explicit state labels for the 12 federal entities with the largest working-equid populations (matching Table 4, Panel A), so no copyrighted base map or third-party imagery is used.

**Reviewer #1**

**Major Concern 1 — Statistical analysis insufficiently rigorous**

***Reviewer comment:*** *The analytical framework relies almost exclusively on the Average Annual Variation Rate (AAVR)... No confidence intervals, sensitivity analyses, or formal significance tests are reported... Analyze the FAOSTAT discrepancy across multiple years rather than only 2022...*

**Response:** A sensitivity analysis was added, testing how the reported population changes would shift under ±5% and ±10% census counting error scenarios (Methods and Table 1). A chi-square test of independence and Cramér's V effect size were added to formally test whether the compositional shift among horses, donkeys, and mules is statistically significant (1970 vs. 2022 and 2007 vs. 2022). The FAOSTAT comparison was extended to all four census years rather than 2022 alone, and FAOSTAT's status flags (official, estimated, imputed) are now reported for each year, showing that the discrepancy grows as the flag shifts from official to imputed. The description of the FAOSTAT comparison as "descriptive statistics" has been corrected.

**Major Concern 2 — IUCN criteria adaptation requires justification**

***Reviewer comment:*** *The authors adapt IUCN Red List decline thresholds to classify federal entities by conservation risk level... applied without adequate justification...*

**Response:** The IUCN-based classification has been removed from the manuscript in its entirety and replaced with the chi-square/Cramér's V analysis described above.

**Major Concern 3 — FAOSTAT discrepancy is analytically incomplete (scope difference)**

***Reviewer comment:*** *INEGI counts only animals registered within active Agricultural Production Units (APUs), while FAOSTAT likely reports total national stock including sport horses, leisure animals, backyard equids... This definitional scope difference could account for a substantial portion of the reported gap.*

**Response:** The Methods section now explicitly defines the Agricultural Production Unit (Unidad de Producción Agropecuaria, UPA) as the census enumeration unit and states that Census counts are restricted to animals recorded within these units. The Discussion addresses the scope difference directly, noting that the census's recreational/sport-use category captures only animals within UPAs and that FAOSTAT's broader national estimates plausibly include animals outside this frame; the finding is now framed as a methodological comparison between two counting frameworks rather than a unidirectional error attributable to FAOSTAT.

**Major Concern 4 — Internal numerical inconsistencies**

***Reviewer comment:*** *The manuscript alternates between describing a "37% recovery" in working equids and a "73% increase" in working horses without consistently clarifying that these refer to different metrics... Table 2 reports the State of Mexico working horse population as 56,125, while a later section states 77,121... Figure 7 caption states a maximum concentration of 104,559...*

**Response:** All figures, tables, and in-text values were recalculated from the source census data and cross-checked for internal consistency during the rewrite. The 37.1% figure now refers specifically and consistently to the total working-equid population (2007–2022), and the 73.0% figure refers specifically and consistently to working horses alone (2007–2022); both are labeled with their specific denominator at every occurrence. The previously inconsistent state-level and figure-caption values no longer appear in this form, as Tables 1–4 and Figures 1–4 were rebuilt directly from the verified INEGI source files rather than carried over from the prior draft.

**Major Concern 5 — Causal language exceeds what the data support**

***Reviewer comment:*** *The "resilience of necessity" explanation... is intellectually compelling but is not supported by any economic data... Similarly, the "paradox of modernization" and "bio-cultural erosion" framings... are inferential narratives rather than data-derived conclusions.*

**Response:** These interpretive framings ("resilience of necessity," "paradox of modernization," "bio-cultural erosion") have been removed. The Discussion now presents the working-horse increase as an association with agricultural mechanization patterns, explicitly described as a hypothesis consistent with the temporal pattern rather than a causally demonstrated mechanism, and the Limitations paragraph states directly that the census data do not allow causal mechanisms to be tested.

**Moderate Concern 6 — Data Availability Statement incomplete**

***Reviewer comment:*** *The submission provides an OSF repository URL but does not include a Data Availability Statement within the manuscript body... no DOI is provided... contents of the repository are not described, no reuse license is specified...*

**Response:** A full Data Availability Statement has been added to the manuscript body (before the Introduction). The OSF repository now contains a fully documented, formula-verified analytical workbook (INEGI census totals, working-equid figures, the sensitivity analysis, the complete 32-federal-entity 2022 breakdown by species, and the FAOSTAT–INEGI comparison) together with the FAOSTAT source CSV. The repository DOI, license, and public-access confirmation have been finalized and are cited in the Data Availability Statement (doi.org/10.17605/OSF.IO/5KAQY); the repository is publicly accessible and no placeholder remains in the manuscript.

**Moderate Concern 7 — Language and tone**

***Reviewer comment:*** *Terms and phrases such as "staggering," "drastic" (used more than six times), "profound contradiction," "relic of the past," "stagnant dogma," "bio-cultural erosion," and "alarming" represent editorial judgments rather than scientific descriptions... key conceptual terms... are introduced without operational definitions...*

**Response:** All flagged evaluative language has been removed during the rewrite; the manuscript now uses neutral, descriptive language throughout (e.g., "decline," "increase," "divergence"). The previously undefined conceptual terms ("statistical invisibility," "paradox of modernization," "resilience of necessity") have been removed along with the interpretive framings they supported, rather than retained and redefined, since the reviewers' concern was that these framings themselves were not fully supported by the data (see Major Concern 5).

**Moderate Concern 8 — Methods section requires strengthening**

***Reviewer comment:*** *Microsoft Excel is identified as the sole analytical tool but no version is reported... No statistical reporting guideline is referenced... the statement that data were obtained from "the Library of the Institute of Geography at UNAM" should clarify whether physical archive access was required for the 1970 census data, and whether these data are reproducibly accessible...*

**Response:** The Statistical Analysis subsection now specifies the software used: Microsoft Excel for Mac, version 16.112.2 (Microsoft 365 subscription). A completed STROBE checklist for cross-sectional studies is now submitted as Supporting Information (S1_STROBE_Checklist). The Methods section clarifies that the 1970 census was published by the Dirección General de Estadística (DGE) as 32 state-level printed volumes, consulted individually at the Library of the Institute of Geography, UNAM, with national totals aggregated manually by the authors; the Limitations paragraph notes that these volumes are reproducibly accessible through the UNAM library or equivalent institutional collections.

**Minor Concern 9 — Table 2 requires redesign**

***Reviewer comment:*** *Table 2... is extremely dense and difficult to read in its current format. The authors should consider splitting this table by region or species, or relocating detailed state-level data to supplementary material...*

**Response:** The tables were restructured during the rewrite. State-level detail is now presented separately from the national time-series tables: Table 4 presents the federal-entity-level detail in three panels rather than all 32 entities in a single dense table: Panel A ranks the 12 federal entities with the largest working-equid populations in 2022, Panel B lists the entities with decreases in the combined working-equid population between 2007 and 2022, and Panel C lists the entities with the largest absolute increases in the horse component of that population. The complete 32-federal-entity breakdown for all three species is provided in full in the OSF-deposited dataset for readers who need it.

**Minor Concern 10 — Map figures need improved legends**

***Reviewer comment:*** *Figures 7, 8, and 9... do not include numerical values that would allow readers to identify state-level figures without cross-referencing Table 2. Adding state labels... would substantially improve figure utility.*

**Response:** The choropleth map figure (Fig 3) now includes state-name labels for the 12 federal entities with the largest working-equid populations, consistent across all three panels (horses, donkeys, mules) and matching Table 4, Panel A. The figure caption states explicitly that only these 12 entities are labeled and that the remaining entities are represented by color only.

**Minor Concern 11 — Horse meat trade section**

***Reviewer comment:*** *The discussion of Mexico's horse meat industry... is presented as explanatory context but is not integrated into the main quantitative analysis... require primary citations...*

**Response:** This section has been removed from the manuscript, as it was not integrated into the quantitative analysis and its inclusion as unintegrated context was the reviewer's specific concern.

**Specific textual corrections**

**Response:** Each of the specific word-level corrections listed by the reviewer ("staggering 710.8%" → "710.8%"; "stagnant dogma" → "persistent assumption"; "relic of the past" → "remnant of a prior agricultural era"; "profound contradiction" → "notable inconsistency"; standardized "APU"/"small-scale" usage; replacement of repeated "drastic") targeted language that has been superseded by the broader language revision described under Moderate Concern 7. The specific words flagged no longer appear anywhere in the manuscript; grammatical corrections noted by the reviewer ("donkeys and mules showed a decline"; "the greatest absolute loss") were incorporated during the Results rewrite.

**Reviewer #2**

***Reviewer comment:*** *The main reason for rejecting the article is the lack of comparison between the methodologies of FAOSTAT and INEGI.*

**Response:** The Introduction now includes a detailed, individually cited description of how each Agricultural Census (1970, 1991, 2007, 2022) defined and recorded equid categories, drawing on the primary census publications (newly added citations for the 1991 and 2007 censuses). The Methods section describes INEGI's field-enumeration approach based on Agricultural Production Units, in contrast with FAOSTAT's own reporting basis and status-flag system (official/estimated/imputed), which is now reported and discussed for every comparison year rather than for 2022 alone.

***Reviewer comment:*** *The statistical analysis remains at a basic level. For example, there is no quantification of the uncertainty in the estimated AAVR.*

**Response:** A sensitivity analysis (±5%/±10% census counting error) was added to quantify how the AAVR-based conclusions would change under plausible measurement uncertainty (Methods; Table 1).

***Reviewer comment:*** *There are also statements made without a clear foundation. One of them refers to the relationship between modernization and the use of working equids. As a suggestion, the authors could include a model relating both quantities.*

**Response:** Rather than adding a formal causal model, which the available census data cannot support (no farm-level mechanization or economic data are collected by the census), we removed the unsupported causal framings this comment refers to (see Reviewer #1, Major Concern 5) and reframed the association between mechanization and working-equid composition as a hypothesis consistent with the temporal pattern, explicitly flagged as such in the Discussion and Limitations.

***Reviewer comment:*** *Another opportunity for the authors is to include the INEGI methodology for the censuses over the years.*

**Response:** This has been added: the Introduction now describes the specific classification structure used in each of the four censuses (1970, 1991, 2007, 2022), each with its own primary citation, and the Methods section describes INEGI's field-enumeration methodology in detail.

**Corrected Funding Statement**

This work was not supported by a specific research grant. E.G.S. received a PASPA fellowship (Programa de Apoyo a la Superación del Personal Académico, DGAPA-UNAM) during the period of this study, as part of doctoral studies. The funder had no role in study design, data collection and analysis, decision to publish, or preparation of the manuscript.

We thank the editor and reviewers again for their time and thorough feedback, which substantially improved the manuscript.
