# Supplementary material for: Equid population dynamics in Mexico over five decades: Divergence between national census records and FAOSTAT estimates
Source: PLoS One. 2026 Nov 5;21(11):e0348247. doi: 10.1371/journal.pone.0348247 (PMC13739109; doi:10.1371/journal.pone.0348247)
Supplement: Peer review history

# Peer review: Decision Letter 0

**DOI:** 10.1371/journal.pone.0348247.r001

**Authors:** Nicolas Padilla-Raygoza

29 Jun 2026

Dear Dr. Toribio,

Thank you for submitting your manuscript to PLOS ONE. After careful consideration, we feel that it has merit but does not fully meet PLOS ONE’s publication criteria as it currently stands. Therefore, we invite you to submit a revised version of the manuscript that addresses the points raised during the review process.

**The authors should take into account all comments from reviewers and correct the manuscript, according to the comments from reviewers, and resubmit the corrected article.** **I believe that, with these changes, the likelihood of accepting the article will increase.**

Please submit your revised manuscript by Aug 13 2026 11:59PM. If you will need more time than this to complete your revisions, please reply to this message or contact the journal office at plosone@plos.org. When you're ready to submit your revision, log on to https://www.editorialmanager.com/pone/ and select the 'Submissions Needing Revision' folder to locate your manuscript file.

- A letter that responds to each point raised by the academic editor and reviewer(s). You should upload this letter as a separate file labeled 'Response to Reviewers'.
- A marked-up copy of your manuscript that highlights changes made to the original version. You should upload this as a separate file labeled 'Revised Manuscript with Track Changes'.
- An unmarked version of your revised paper without tracked changes. You should upload this as a separate file labeled 'Manuscript'.

If you would like to make changes to your financial disclosure, please include your updated statement in your cover letter. Guidelines for resubmitting your figure files are available below the reviewer comments at the end of this letter.

If applicable, we recommend that you deposit your laboratory protocols in protocols.io to enhance the reproducibility of your results. Protocols.io assigns your protocol its own identifier (DOI) so that it can be cited independently in the future. For instructions see: https://journals.plos.org/plosone/s/submission-guidelines#loc-laboratory-protocols. Additionally, PLOS ONE offers an option for publishing peer-reviewed Lab Protocol articles, which describe protocols hosted on protocols.io. Read more information on sharing protocols at https://plos.org/protocols?utm_medium=editorial-email&utm_source=authorletters&utm_campaign=protocols.

As the corresponding author, your ORCID iD is verified in the submission system and will appear in the published article. PLOS supports the use of ORCID, and we encourage all coauthors to register for an ORCID iD and use it as well. Please encourage your coauthors to verify their ORCID iD within the submission system before final acceptance, as unverified ORCID iDs will not appear in the published article. *Only*  the individual author can complete the verification step; PLOS staff *cannot*  verify ORCID iDs on behalf of authors.

We look forward to receiving your revised manuscript.

Kind regards,

Nicolas Padilla-Raygoza, Doctorate in Sciences

Academic Editor

PLOS One

Journal Requirements:

When submitting your revision, we need you to address these additional requirements.

1. Please ensure that your manuscript meets PLOS ONE’s style requirements, including those for file naming. The PLOS ONE style templates can be found at

https://journals.plos.org/plosone/s/file?id=wjVg/PLOSOne_formatting_sample_main_body.pdf and

https://journals.plos.org/plosone/s/file?id=ba62/PLOSOne_formatting_sample_title_authors_affiliations.pdf

2. Please note that PLOS One has specific guidelines on code sharing for submissions in which author-generated code underpins the findings in the manuscript. In these cases, we expect all author-generated code to be made available without restrictions upon publication of the work. Please review our guidelines at https://journals.plos.org/plosone/s/materials-and-software-sharing#loc-sharing-code and ensure that your code is shared in a way that follows best practice and facilitates reproducibility and reuse.

3. Thank you for stating the following in the Acknowledgments Section of your manuscript:

"UNAM Students: Luis Eduardo MolinaCorona, Javier Beltran Romero, Erick Zuñiga Martínez, and the students from the Equine Specialty Program. This study was partially funded by the Universidad Nacional Autónoma de México (UNAM) through the DGAPA-PAPIIT project IN220424 during its first year. The funding institution had no role in study design, data collection and analysis, decision to publish, or preparation of the manuscript."

We note that you have provided additional information within the Acknowledgements Section that is not currently declared in your Funding Statement. Please note that funding information should not appear in the Acknowledgments section or other areas of your manuscript. We will only publish funding information present in the Funding Statement section of the online submission form.

Please remove any funding-related text from the manuscript and let us know how you would like to update your Funding Statement. Currently, your Funding Statement reads as follows:

"EGS.

Grant #: IN220424.

Funder: Universidad Nacional Autónoma de México (UNAM).

The funding institution had no role in study design, data collection and analysis, decision to publish, or preparation of the manuscript. "

Please include your amended statements within your cover letter; we will change the online submission form on your behalf.

4. We note that Figures 1, 7, 8 and 9 in your submission contain map images which may be copyrighted. All PLOS content is published under the Creative Commons Attribution License (CC BY 4.0), which means that the manuscript, images, and Supporting Information files will be freely available online, and any third party is permitted to access, download, copy, distribute, and use these materials in any way, even commercially, with proper attribution. For these reasons, we cannot publish previously copyrighted maps or satellite images created using proprietary data, such as Google software (Google Maps, Street View, and Earth). For more information, see our copyright guidelines: http://journals.plos.org/plosone/s/licenses-and-copyright.

We require you to either (1) present written permission from the copyright holder to publish these figures specifically under the CC BY 4.0 license, or (2) remove the figures from your submission:

1. You may seek permission from the original copyright holder of Figures 1, 7, 8 and 9 to publish the content specifically under the CC BY 4.0 license.

We recommend that you contact the original copyright holder with the Content Permission Form (http://journals.plos.org/plosone/s/file?id=7c09/content-permission-form.pdf) and the following text:

“I request permission for the open-access journal PLOS ONE to publish XXX under the Creative Commons Attribution License (CCAL) CC BY 4.0 (http://creativecommons.org/licenses/by/4.0/). Please be aware that this license allows unrestricted use and distribution, even commercially, by third parties. Please reply and provide explicit written permission to publish XXX under a CC BY license and complete the attached form.”

Please upload the completed Content Permission Form or other proof of granted permissions as an "Other" file with your submission.

In the figure caption of the copyrighted figure, please include the following text: “Reprinted from [ref] under a CC BY license, with permission from [name of publisher], original copyright [original copyright year].”

2. If you are unable to obtain permission from the original copyright holder to publish these figures under the CC BY 4.0 license or if the copyright holder’s requirements are incompatible with the CC BY 4.0 license, please either i) remove the figure or ii) supply a replacement figure that complies with the CC BY 4.0 license. Please check copyright information on all replacement figures and update the figure caption with source information. If applicable, please specify in the figure caption text when a figure is similar but not identical to the original image and is therefore for illustrative purposes only.

The following resources for replacing copyrighted map figures may be helpful:

USGS National Map Viewer (public domain): http://viewer.nationalmap.gov/viewer/

The Gateway to Astronaut Photography of Earth (public domain): http://eol.jsc.nasa.gov/sseop/clickmap/

Maps at the CIA (public domain): https://www.cia.gov/library/publications/the-world-factbook/index.html and https://www.cia.gov/library/publications/cia-maps-publications/index.html

NASA Earth Observatory (public domain): http://earthobservatory.nasa.gov/

Landsat: http://landsat.visibleearth.nasa.gov/

USGS EROS (Earth Resources Observatory and Science (EROS) Center) (public domain): http://eros.usgs.gov/#

Natural Earth (public domain): http://www.naturalearthdata.com/

5. If the reviewer comments include a recommendation to cite specific previously published works, please review and evaluate these publications to determine whether they are relevant and should be cited. There is no requirement to cite these works unless the editor has indicated otherwise.

Additional Editor Comments :

I consider that the authors should take into account all comments from reviewers to increase the quality of the article.

[Note: HTML markup is below. Please do not edit.]

Reviewers' comments:

Reviewer’s Responses to Questions

**Comments to the Author**

1. Is the manuscript technically sound, and do the data support the conclusions?

Reviewer #1: Partly

Reviewer #2: No

**********

2. Has the statistical analysis been performed appropriately and rigorously?

Reviewer #1: N/A

Reviewer #2: No

**********

3. Have the authors made all data underlying the findings in their manuscript fully available?

The PLOS Data policy

Reviewer #1: No

Reviewer #2: Yes

**********

4. Is the manuscript presented in an intelligible fashion and written in standard English?

Reviewer #1: Yes

Reviewer #2: Yes

**********

Reviewer #1: Summary

This manuscript addresses an important and underexplored topic: the population dynamics of working equids in Mexico over a 52-year period and the substantial discrepancy between national census data and international estimates. The subject matter is relevant to veterinary epidemiology, rural development policy, and global livestock monitoring. The authors are commended for assembling a longitudinal dataset spanning four census points and for identifying a genuinely striking data discrepancy between INEGI and FAOSTAT records. However, the manuscript requires substantial revision in statistical rigor, methodological justification, internal consistency, language quality, and data availability compliance before it can be considered for publication in PLOS ONE.

Major Concerns

1. Statistical Analysis Is Insufficiently Rigorous

The analytical framework relies almost exclusively on the Average Annual Variation Rate (AAVR) and arithmetic comparison. While the AAVR is correctly applied, the manuscript presents all estimates as precise point values without any uncertainty quantification. No confidence intervals, sensitivity analyses, or formal significance tests are reported. For a study making comparative claims across species, time periods, and geographic regions, this represents a critical gap.

Specifically, the authors should:

Report uncertainty ranges around all AAVR estimates to reflect the potential for census counting error

Conduct at minimum a chi-square test of proportions to formally establish whether the compositional shift from donkeys and mules toward horses is statistically significant

Perform a sensitivity analysis testing how conclusions would change under plausible census error scenarios of ±5% and ±10%

Analyze the FAOSTAT discrepancy across multiple years rather than only 2022, to determine whether the gap is stable, growing, or a recent artifact

The manuscript states that "descriptive statistics" were used for the FAOSTAT comparison, but the actual computation is arithmetic subtraction. This characterization overstates the analytical depth applied to the study’s headline finding.

2. The IUCN Criteria Adaptation Requires Formal Justification

The authors adapt IUCN Red List decline thresholds to classify federal entities by conservation risk level. This is a creative methodological choice, but it is applied without adequate justification. The original IUCN criteria were designed for wild species facing extinction risk over biologically grounded time windows of 10 years or three generations. The adaptation here uses a 15-year window determined by census availability and applies thresholds — 10%, 30%, 50%, 70% — for which no statistical, biological, or precedent-based rationale is provided.

The authors must either: (a) provide explicit derivation or precedent citations for each threshold level; (b) cite prior peer-reviewed studies that have applied comparable adaptations to domestic animals; or (c) significantly moderate the language around these classifications, presenting them as descriptive categories rather than validated conservation assessments. The current presentation risks misleading readers about the formal conservation status of these animal populations.

3. The FAOSTAT Discrepancy Finding Is Arithmetically Correct but Analytically Incomplete

The 710.8% discrepancy is the manuscript’s most prominent finding, yet its interpretation is underdeveloped. The authors characterize the difference as an "overestimation" by FAO without adequately addressing the possibility that FAOSTAT and INEGI measure different populations. INEGI counts only animals registered within active Agricultural Production Units (APUs), while FAOSTAT likely reports total national stock including sport horses, leisure animals, backyard equids, and animals not affiliated with formal agricultural operations. This definitional scope difference could account for a substantial portion of the reported gap.

The authors should: formally document FAO’s estimation methodology for Mexico; explicitly address scope differences between the two counting frameworks; quantify, if possible, the population of equids outside APUs that INEGI does not capture; and reframe the finding as a methodological comparison rather than a unidirectional error attribution. The current framing overstates the conclusion the data can support.

4. Internal Numerical Inconsistencies Must Be Resolved

Several numerical discrepancies appear across the manuscript that undermine confidence in the accuracy of the reported figures:

The manuscript alternates between describing a "37% recovery" in working equids and a "73% increase" in working horses without consistently clarifying that these refer to different metrics. This confusion appears in both the abstract and results sections and must be resolved with clear, consistent labeling throughout.

Table 2 reports the State of Mexico working horse population as 56,125, while a later section of the manuscript states 77,121 for the same entity and year. This discrepancy must be explained or corrected.

Figure 7 caption states a maximum concentration of 104,559 working horses, which is not reconcilable with the figures in Table 2 without explanation.

All figures, tables, and text must be cross-checked for internal consistency prior to resubmission.

5. Causal Language Exceeds What the Data Support

The manuscript presents several interpretive conclusions as if they were empirically established findings. The "resilience of necessity" explanation — attributing the horse population recovery to rising fuel costs and lack of rural credit — is intellectually compelling but is not supported by any economic data presented in the manuscript. Similarly, the "paradox of modernization" and "bio-cultural erosion" framings, while evocative, are inferential narratives rather than data-derived conclusions.

The authors should either: present economic data on fuel prices and rural credit access to support these claims; or reframe these interpretations explicitly as hypotheses or proposed mechanisms warranting future empirical investigation, rather than conclusions of the present study.

Moderate Concerns

6. Data Availability Statement Is Incomplete

The submission provides an OSF repository URL (https://osf.io/5kaqy/overview) but does not include a Data Availability Statement within the manuscript body, as required by PLOS ONE policy. Additionally, no DOI is provided for the repository, the contents of the repository are not described, no reuse license is specified, and it cannot be verified from the submission whether the repository is publicly accessible at the time of submission.

The authors must: add a formal Data Availability Statement to the manuscript body; register a DOI for the OSF repository; describe the repository contents; specify the applicable reuse license; and confirm public accessibility. The compiled analytical dataset — including organized census figures, AAVR calculations, IUCN classifications by federal entity, and the FAOSTAT comparison table — should be deposited even though the underlying government census data are publicly available, to ensure reproducibility.

7. Language and Tone Require Revision

The manuscript is written in intelligible English but employs evaluative and emotive language inconsistent with the neutral scientific register expected in a PLOS ONE research article. Terms and phrases such as "staggering," "drastic" (used more than six times), "profound contradiction," "relic of the past," "stagnant dogma," "bio-cultural erosion," and "alarming" represent editorial judgments rather than scientific descriptions. These should be replaced with neutral, precise language throughout.

Additionally, key conceptual terms — "statistical invisibility," "paradox of modernization," and "resilience of necessity" — are introduced without operational definitions and are used in subtly different senses across sections. Each of these terms must be formally defined at its first occurrence and used consistently thereafter.

The authors are encouraged to engage a professional scientific English editing service prior to resubmission. PLOS ONE provides a list of recommended language editing services on its website.

8. Methods Section Requires Strengthening

The Methods section omits several details necessary for reproducibility and transparency:

Microsoft Excel is identified as the sole analytical tool but no version is reported. The version should be specified.

The IUCN threshold levels are listed without narrative justification, as discussed above.

No statistical reporting guideline is referenced. For an observational epidemiological study of this design, the STROBE checklist would be most appropriate and should be completed and submitted as supplementary material.

The statement that data were obtained from "the Library of the Institute of Geography at UNAM" should clarify whether physical archive access was required for the 1970 census data, and whether these data are reproducibly accessible to other researchers.

Minor Concerns

9. Table 2 Requires Redesign

Table 2, presenting regional distribution and population dynamics across all federal entities, species, and census years, is extremely dense and difficult to read in its current format. The authors should consider splitting this table by region or species, or relocating detailed state-level data to supplementary material with a condensed summary table in the main text. The current format presents a barrier to comprehension that will disadvantage the manuscript with readers and reviewers alike.

10. Map Figures Need Improved Legends

Figures 7, 8, and 9 use choropleth shading to represent working equid populations by state but do not include numerical values that would allow readers to identify state-level figures without cross-referencing Table 2. Adding state labels or an interactive legend, or at minimum indicating the values corresponding to the darkest and lightest shading categories, would substantially improve figure utility.

11. The Horse Meat Trade Section Needs Integration or Relocation

The discussion of Mexico’s horse meat industry — including figures for imported US horses and export volumes to Europe and Japan — is presented as explanatory context but is not integrated into the main quantitative analysis. The figures cited (400,000 animals processed annually, 70,000 tons of meat) require primary citations, and the relationship between the meat trade and working equid population trends should be either formally analyzed or clearly identified as a proposed mechanism for future investigation rather than a confirmed explanatory factor.

Specific Textual Corrections

The following specific revisions are recommended in addition to the broader concerns above:

Abstract: Replace "a staggering 710.8% discrepancy" with "a 710.8% discrepancy"

Introduction: Replace "stagnant dogma" with "persistent assumption"

Results, p.8: Correct "donkeys and mules show a decline" to "donkeys and mules showed a decline"

Results, p.9: Correct "the most absolute loss" to "the greatest absolute loss"

Discussion: Replace "relic of the past" with "remnant of a prior agricultural era"

Discussion: Replace "profound contradiction" with "notable inconsistency"

Throughout: Standardize "APU" definition and usage; standardize hyphenation of "small-scale"

Throughout: Replace repeated use of "drastic" with varied alternatives such as "substantial," "marked," or "severe"

Conclusion

This manuscript makes a genuine contribution to the literature on working animal populations and international data discrepancies in livestock monitoring. The longitudinal dataset and the identification of the FAOSTAT gap are valuable. With substantial revision addressing the statistical, methodological, interpretive, and presentational concerns outlined above, this work has the potential to be a meaningful addition to the field. The authors are encouraged to undertake a thorough revision and to consider independent statistical consultation before resubmission.

Reviewer #2: Although it is a very sound topic, there are major concerns about the methodology. The main reason for rejecting the article is the lack of comparison between the methodologies of FAOSTAT and INEGI. Besides, the statistical analysis remains at a basic level. For example, there is no quantification of the uncertainty in the estimated AAVR. There are also statements made without a clear foundation. One of them refers to the relationship between modernization and the use of working equids. As a suggestion, the authors could include a model relating both quantities. Another opportunity for the authors is to include the INEGI methodology for the censuses over the years.

**********

what does this mean?). If published, this will include your full peer review and any attached files.

If you choose “no”, your identity will remain anonymous but your review may still be made public.

**Do you want your identity to be public for this peer review?**  For information about this choice, including consent withdrawal, please see our Privacy Policy

Reviewer #1: **Yes:**  Yahya Ali Abdulkareem Abodea

Reviewer #2: No

**********

[NOTE: If reviewer comments were submitted as an attachment file, they will be attached to this email and accessible via the submission site. Please log into your account, locate the manuscript record, and check for the action link "View Attachments". If this link does not appear, there are no attachment files.]

To ensure your figures meet our technical requirements, please review our figure guidelines: https://journals.plos.org/plosone/s/figures

You may also use PLOS’s free figure tool, NAAS, to help you prepare publication quality figures: https://journals.plos.org/plosone/s/figures#loc-tools-for-figure-preparation.

NAAS will assess whether your figures meet our technical requirements by comparing each figure against our figure specifications.

# Author comment: Author response to Decision Letter 1

**DOI:** 10.1371/journal.pone.0348247.r002

14 Sep 2026

We have revised the entire manuscript, reduced the number of figures, and provide our responses to the reviewers.

Response to Reviewers

Manuscript: "Equid population dynamics in Mexico over five decades: Divergence between national census records and FAOSTAT estimates"

We thank the Academic Editor and both reviewers for their careful and constructive evaluation of our manuscript. In response to their comments, the manuscript has been substantially rewritten. Below we summarize the scope of that revision, then respond point by point to every comment raised in the review. A version of the manuscript with tracked changes against the originally submitted version is included as a separate file with this resubmission.

Summary of the revision

Because the reviewers' concerns touched on nearly every part of the manuscript — statistical rigor, methodological justification, internal consistency, data availability, and language — we chose to rewrite the manuscript rather than patch it incrementally. The principal changes are:

● The title and framing were changed from an evaluative framing ("Statistical invisibility of working equids in Mexico...") to a neutral, descriptive one ("Equid population dynamics in Mexico over five decades: Divergence between national census records and FAOSTAT estimates").

● The adapted IUCN Red List classification was removed in its entirety and replaced with a formal chi-square test of independence and Cramér’s V effect size for compositional change, plus a sensitivity analysis (±5%/±10%) addressing census counting error.

● The FAOSTAT–INEGI comparison was extended from a single year (2022) to all four census years (1970, 1991, 2007, 2022), and FAOSTAT’s own data-status flags (official/estimated/imputed) are now reported and discussed.

● All causal and evaluative language flagged by Reviewer #1 ("staggering," "drastic," "resilience of necessity," "paradox of modernization," "bio-cultural erosion," etc.) was removed; undefined conceptual terms were either formally defined or eliminated.

● The horse-meat-trade section was removed, as it was not integrated into the quantitative analysis.

● A formal Data Availability Statement was added to the manuscript body, an OSF repository was prepared with the full analytical dataset (including the complete 32-federal-entity 2022 breakdown), and a STROBE checklist is now submitted as supplementary material (S1).

● All in-text citations were converted to PLOS ONE’s numbered format, and the reference list was expanded and re-verified, including the addition of primary citations for the 1991 and 2007 Agricultural Censuses.

● The choropleth map figure was rebuilt from non-copyrighted boundary data obtained directly from the INEGI Marco Geoestadístico, with correct federal-entity labels, replacing the original figures flagged for possible copyright issues.

● Funding information was removed from the Acknowledgments section; the corrected Funding Statement is provided at the end of this letter.

Because of the scope of this rewrite, several of the reviewers' line-level textual corrections (e.g., specific word replacements in since-removed sentences) no longer apply to specific sentences — the underlying language issue they identify has been resolved throughout the manuscript rather than at the original location. We note this explicitly where relevant below.

Editor / Journal Requirements

Reviewer comment: Thank you for stating funding information in the Acknowledgments Section... Please remove any funding-related text from the manuscript and let us know how you would like to update your

Funding Statement.

Response: The funding-related text has been removed from the Acknowledgments section, which now lists only the student contributors (Luis Eduardo Molina Corona, Javier Beltrán Romero, Erick Zuñiga Martínez, and María Fátima Rodríguez Vargas). The corrected Funding Statement, to be entered in the online submission system, is: "This work was not supported by a specific research grant. E.G.S. received a PASPA fellowship (Programa de Apoyo a la Superación del Personal Académico, DGAPA-UNAM) during the period of this study, as part of doctoral studies. The funder had no role in study design, data collection and analysis, decision to publish, or preparation of the manuscript."

Reviewer comment: We note that Figures 1, 7, 8 and 9 in your submission contain map images which may be copyrighted... We require you to either (1) present written permission from the copyright holder... or (2) remove the figures.

Response: The choropleth map figure was rebuilt using state boundaries obtained directly from INEGI’s own Marco Geoestadístico (https://www.inegi.org.mx/programas/mg/), the same official geographic boundary dataset cited in the manuscript’s Materials and Methods, not from any proprietary or copyrighted map service. The figure now includes explicit state labels for the 12 federal entities with the largest working-equid populations (matching Table 4, Panel A), so no copyrighted base map or third-party imagery is used.

Reviewer #1

Major Concern 1 — Statistical analysis insufficiently rigorous

Reviewer comment: The analytical framework relies almost exclusively on the Average Annual Variation Rate (AAVR)... No confidence intervals, sensitivity analyses, or formal significance tests are reported... Analyze the FAOSTAT discrepancy across multiple years rather than only 2022...

Response: A sensitivity analysis was added, testing how the reported population changes would shift under ±5% and ±10% census counting error scenarios (Methods and Table 1). A chi-square test of independence and Cramér’s V effect size were added to formally test whether the compositional shift among horses, donkeys, and mules is statistically significant (1970 vs. 2022 and 2007 vs. 2022). The FAOSTAT comparison was extended to all four census years rather than 2022 alone, and FAOSTAT’s status flags (official, estimated, imputed) are now reported for each year, showing that the discrepancy grows as the flag shifts from official to imputed. The description of the FAOSTAT comparison as "descriptive statistics" has been corrected.

Major Concern 2 — IUCN criteria adaptation requires justification

Reviewer comment: The authors adapt IUCN Red List decline thresholds to classify federal entities by conservation risk level... applied without adequate justification...

Response: The IUCN-based classification has been removed from the manuscript in its entirety and replaced with the chi-square/Cramér’s V analysis described above.

Major Concern 3 — FAOSTAT discrepancy is analytically incomplete (scope difference)

Reviewer comment: INEGI counts only animals registered within active Agricultural Production Units (APUs), while FAOSTAT likely reports total national stock including sport horses, leisure animals, backyard equids... This definitional scope difference could account for a substantial portion of the reported gap.

Response: The Methods section now explicitly defines the Agricultural Production Unit (Unidad de Producción Agropecuaria, UPA) as the census enumeration unit and states that Census counts are restricted to animals recorded within these units. The Discussion addresses the scope difference directly, noting that the census’s recreational/sport-use category captures only animals within UPAs and that FAOSTAT’s broader national estimates plausibly include animals outside this frame; the finding is now framed as a methodological comparison between two counting frameworks rather than a unidirectional error attributable to FAOSTAT.

Major Concern 4 — Internal numerical inconsistencies

Reviewer comment: The manuscript alternates between describing a "37% recovery" in working equids and a "73% increase" in working horses without consistently clarifying that these refer to different metrics... Table 2 reports the State of Mexico working horse population as 56,125, while a later section states 77,121... Figure 7 caption states a maximum concentration of 104,559...

Response: All figures, tables, and in-text values were recalculated from the source census data and cross-checked for internal consistency during the rewrite. The 37.1% figure now refers specifically and consistently to the total working-equid population (2007–2022), and the 73.0% figure refers specifically and consistently to working horses alone (2007–2022); both are labeled with their specific denominator at every occurrence. The previously inconsistent state-level and figure-caption values no longer appear in this form, as Tables 1–4 and Figures 1–4 were rebuilt directly from the verified INEGI source files rather than carried over from the prior draft.

Major Concern 5 — Causal language exceeds what the data support

Reviewer comment: The "resilience of necessity" explanation... is intellectually compelling but is not supported by any economic data... Similarly, the "paradox of modernization" and "bio-cultural erosion" framings... are inferential narratives rather than data-derived conclusions.

Response: These interpretive framings ("resilience of necessity," "paradox of modernization," "bio-cultural erosion") have been removed. The Discussion now presents the working-horse increase as an association with agricultural mechanization patterns, explicitly described as a hypothesis consistent with the temporal pattern rather than a causally demonstrated mechanism, and the Limitations paragraph states directly that the census data do not allow causal mechanisms to be tested.

Moderate Concern 6 — Data Availability Statement incomplete

Reviewer comment: The submission provides an OSF repository URL but does not include a Data Availability Statement within the manuscript body... no DOI is provided... contents of the repository are not described, no reuse license is specified...

Response: A full Data Availability Statement has been added to the manuscript body (before the Introduction). The OSF repository now contains a fully documented, formula-verified analytical workbook (INEGI census totals, working-equid figures, the sensitivity analysis, the complete 32-federal-entity 2022 breakdown by species, and the FAOSTAT–INEGI comparison) together with the FAOSTAT source CSV. The repository DOI, license, and public-access confirmation have been finalized and are cited in the Data Availability Statement (doi.org/10.17605/OSF.IO/5KAQY); the repository is publicly accessible and no placeholder remains in the manuscript.

Moderate Concern 7 — Language and tone

Reviewer comment: Terms and phrases such as "staggering," "drastic" (used more than six times), "profound contradiction," "relic of the past," "stagnant dogma," "bio-cultural erosion," and "alarming" represent editorial judgments rather than scientific descriptions... key conceptual terms... are introduced without operational definitions...

Response: All flagged evaluative language has been removed during the rewrite; the manuscript now uses neutral, descriptive language throughout (e.g., "decline," "increase," "divergence"). The previously undefined conceptual terms ("statistical invisibility," "paradox of modernization," "resilience of necessity") have been removed along with the interpretive framings they supported, rather than retained and redefined, since the reviewers' concern was that these framings themselves were not fully supported by the data (see Major Concern 5).

Moderate Concern 8 — Methods section requires strengthening

Reviewer comment: Microsoft Excel is identified as the sole analytical tool but no version is reported... No statistical reporting guideline is referenced... the statement that data were obtained from "the Library of the Institute of Geography at UNAM" should clarify whether physical archive access was required for the 1970 census data, and whether these data are reproducibly accessible...

Response: The Statistical Analysis subsection now specifies the software used: Microsoft Excel for Mac, version 16.112.2 (Microsoft 365 subscription). A completed STROBE checklist for cross-sectional studies is now submitted as Supporting Information (S1_STROBE_Checklist). The Methods section clarifies that the 1970 census was published by the Dirección General de Estadística (DGE) as 32 state-level printed volumes, consulted individually at the Library of the Institute of Geography, UNAM, with national totals aggregated manually by the authors; the Limitations paragraph notes that these volumes are reproducibly accessible through the UNAM library or equivalent institutional collections.

Minor Concern 9 — Table 2 requires redesign

Reviewer comment: Table 2... is extremely dense and difficult to read in its current format. The authors should consider splitting this table by region or species, or relocating detailed state-level data to supplementary material...

Response: The tables were restructured during the rewrite. State-level detail is now presented separately from the national time-series tables: Table 4 presents the federal-entity-level detail in three panels rather than all 32 entities in a single dense table: Panel A ranks the 12 federal entities with the largest working-equid populations in 2022, Panel B lists the entities with decreases in the combined working-equid population between 2007 and 2022, and Panel C lists the entities with the largest absolute increases in the horse component of that population. The complete 32-federal-entity breakdown for all three species is provided in full in the OSF-deposited dataset for readers who need it.

Minor Concern 10 — Map figures need improved legends

Reviewer comment: Figures 7, 8, and 9... do not include numerical values that would allow readers to identify state-level figures without cross-referencing Table 2. Adding state labels... would substantially improve figure utility.

Response: The choropleth map figure (Fig 3) now includes state-name labels for the 12 federal entities with the largest working-equid populations, consistent across all three panels (horses, donkeys, mules) and matching Table 4, Panel A. The figure caption states explicitly that only these 12 entities are labeled and that the remaining entities are represented by color only.

Minor Concern 11 — Horse meat trade section

Reviewer comment: The discussion of Mexico’s horse meat industry... is presented as explanatory context but is not integrated into the main quantitative analysis... require primary citations...

Response: This section has been removed from the manuscript, as it was not integrated into the quantitative analysis and its inclusion as unintegrated context was the reviewer’s specific concern.

Specific textual corrections

Response: Each of the specific word-level corrections listed by the reviewer ("staggering 710.8%" → "710.8%"; "stagnant dogma" → "persistent assumption"; "relic of the past" → "remnant of a prior agricultural era"; "profound contradiction" → "notable inconsistency"; standardized "APU"/"small-scale" usage; replacement of repeated "drastic") targeted language that has been superseded by the broader language revision described under Moderate Concern 7. The specific words flagged no longer appear anywhere in the manuscript; grammatical corrections noted by the reviewer ("donkeys and mules showed a decline"; "the greatest absolute loss") were incorporated during the Results rewrite.

Reviewer #2

Reviewer comment: The main reason for rejecting the article is the lack of comparison between the methodologies of FAOSTAT and INEGI.

Response: The Introduction now includes a detailed, individually cited description of how each Agricultural Census (1970, 1991, 2007, 2022) defined and recorded equid categories, drawing on the primary census publications (newly added citations for the 1991 and 2007 censuses). The Methods section describes INEGI’s field-enumeration approach based on Agricultural Production Units, in contrast with FAOSTAT’s own reporting basis and status-flag system (official/estimated/imputed), which is now reported and discussed for every comparison year rather than for 2022 alone.

Reviewer comment: The statistical analysis remains at a basic level. For example, there is no quantification of the uncertainty in the estimated AAVR.

Response: A sensitivity analysis (±5%/±10% census counting error) was added to quantify how the AAVR-based conclusions would change under plausible measurement uncertainty (Methods; Table 1).

Reviewer comment: There are also statements made without a clear foundation. One of them refers to the relationship between modernization and the use of working equids. As a suggestion, the authors could include a model relating both quantities.

Response: Rather than adding a formal causal model, which the available census data cannot support (no farm-level mechanization or economic data are collected by the census), we removed the unsupported causal framings this comment refers to (see Reviewer #1, Major Concern 5) and reframed the association between mechanization and working-equid composition as a hypothesis consistent with the temporal pattern, explicitly flagged as such in the Discussion and Limitations.

Reviewer comment: Another opportunity for the authors is to include the INEGI methodology for the censuses over the years.

Response: This has been added: the Introduction now describes the specific classification structure used in each of the four censuses (1970, 1991, 2007, 2022), each with its own primary citation, and the Methods section describes INEGI’s field-enumeration methodology in detail.

Corrected Funding Statement

This work was not supported by a specific research grant. E.G.S. received a PASPA fellowship (Programa de Apoyo a la Superación del Personal Académico, DGAPA-UNAM) during the period of this study, as part of doctoral studies. The funder had no role in study design, data collection and analysis, decision to publish, or preparation of the manuscript.

We thank the editor and reviewers again for their time and thorough feedback, which substantially improved the manuscript.

Attachment Submitted filename: Response_to_Reviewers (1).docx

# Editor report: Decision Letter 1

**DOI:** 10.1371/journal.pone.0348247.r003

**Authors:** Nicolas Padilla-Raygoza

14 Oct 2026

Equid population dynamics in Mexico over five decades: Divergence between national census records and FAOSTAT estimates.

PONE-D-26-17945R1

Dear Dr. Toribio,

We’re pleased to inform you that your manuscript has been judged scientifically suitable for publication and will be formally accepted for publication once it meets all outstanding technical requirements.

Within one week, you’ll receive an e-mail detailing the required amendments. When these have been addressed, you’ll receive a formal acceptance letter and your manuscript will be scheduled for publication.

An invoice will be generated when your article is formally accepted. Please note, if your institution has a publishing partnership with PLOS and your article meets the relevant criteria, all or part of your publication costs will be covered. Please make sure your user information is up-to-date by logging into Editorial Manager at Editorial Manager® and clicking the ‘Update My Information' link at the top of the page. For questions related to billing, please contact billing support.

If your institution or institutions have a press office, please notify them about your upcoming paper to help maximize its impact. If they’ll be preparing press materials, please inform our press team as soon as possible -- no later than 48 hours after receiving the formal acceptance. Your manuscript will remain under strict press embargo until 2 pm Eastern Time on the date of publication. For more information, please contact onepress@plos.org.

Kind regards,

Nicolas Padilla-Raygoza, Doctorate in Sciences

Academic Editor

PLOS One

Additional Editor Comments (optional):

You corrected the article based on the comments from reviewer, because of this, I consider that the article should be accept.

Reviewers' comments:

# Editor report: Acceptance letter

**DOI:** 10.1371/journal.pone.0348247.r004

**Authors:** Nicolas Padilla-Raygoza

PONE-D-26-17945R1

PLOS One

Dear Dr. Toribio,

I'm pleased to inform you that your manuscript has been deemed suitable for publication in PLOS One. Congratulations! Your manuscript is now being handed over to our production team.

At this stage, our production department will prepare your paper for publication. This includes ensuring the following:

* All references, tables, and figures are properly cited

* All relevant supporting information is included in the manuscript submission,

* There are no issues that prevent the paper from being properly typeset

You will receive further instructions from the production team, including instructions on how to review your proof when it is ready. Please keep in mind that we are working through a large volume of accepted articles, so please give us a few days to review your paper and let you know the next and final steps.

Lastly, if your institution or institutions have a press office, please let them know about your upcoming paper now to help maximize its impact. If they'll be preparing press materials, please inform our press team within the next 48 hours. Your manuscript will remain under strict press embargo until 2 pm Eastern Time on the date of publication. For more information, please contact onepress@plos.org.

You will receive an invoice from PLOS for your publication fee after your manuscript has reached the completed accept phase. If you receive an email requesting payment before acceptance or for any other service, this may be a phishing scheme. Learn how to identify phishing emails and protect your accounts at https://explore.plos.org/phishing.

If we can help with anything else, please email us at customercare@plos.org.

Thank you for submitting your work to PLOS One and supporting open access.

Kind regards,

PLOS One Editorial Office Staff

on behalf of

Dr. Nicolas Padilla-Raygoza

Academic Editor

PLOS One
